# Prediction of Preterm Delivery Using Serum Ischemia Modified Albumin, Biglycan, and Decorin Levels in Women with Threatened Preterm Labor

**DOI:** 10.1055/s-0043-1772593

**Published:** 2023-12-23

**Authors:** Ismail Biyik, Cenk Soysal, Ozlem Ulas Onur Ince, Sinem Durmus, Efser Oztas, Nadi Keskin, Ozben Ozden Isiklar, Oğuz Han Karaagac, Remise Gelisgen, Hafize Uzun

**Affiliations:** 1 Department of Obstetrics and Gynecology, School of Medicine, Kutahya Health Sciences University, Kutahya, Turkey; 2 Department of Statistics, Faculty of Arts and Sciences, Middle East Technical University, Ankara, Turkey; 3 Department of Medical Biochemistry, School of Medicine, Istanbul University-Cerrahpasa, Istanbul, Turkey; 4 Department of Medical Biochemistry, School of Medicine, Kutahya Health Sciences University, Kutahya, Turkey; 5 Department of Medical Biochemistry, Faculty of Medicine, Istanbul Atlas University, Istanbul, Turkey

**Keywords:** ischemia modified albumin, biglycan decorin, preterm delivery prediction, threatened preterm labor, preterm delivery, albumina modificada por isquemia, biglicano decorina, previsão de parto prematuro, ameaça de parto prematuro, parto prematuro

## Abstract

**Objective** The serum ischemia modified albumin (IMA), biglycan, and decorin levels of pregnant women who were hospitalized for threatened preterm labor were measured.

**Methods** Fifty-one consecutive pregnant women with a single pregnancy between the 24 ^th^ and 36 ^th^ weeks with a diagnosis of threatened preterm labor were included in the present prospective cohort study.

**Results** As a result of multivariate logistic regression analysis for predicting preterm delivery within 24 hours, 48 hours, 7 days, 14 days, ≤ 35 gestational weeks, and ≤ 37 gestational weeks after admission, area under the curve (AUC) (95% confidence interval [CI[) values were 0.95 (0.89–1.00), 0.93 (0.86–0.99), 0.91 (0.83–0.98), 0.92 (0.85–0.99), 0.82 (0.69–0.96), and 0.89 (0.80–0.98), respectively. In the present study, IMA and biglycan levels were found to be higher and decorin levels lower in women admitted to the hospital with threatened preterm labor and who gave preterm birth within 48 hours compared with those who gave birth after 48 hours.

**Conclusion** In pregnant women admitted to the hospital with threatened preterm labor, the prediction preterm delivery of the combined model created by adding IMA, decorin, and biglycan in addition to the TVS CL measurement was higher than the TVS CL measurement alone.

**Clinical trial registration** The present trial was registered at ClinicalTrials.gov, number NCT04451928.

## Resumo

**Objetivo** Medir os níveis séricos de albumina modificada por isquemia (IMA), biglicano e decorina de gestantes hospitalizadas por ameaça de parto prematuro.

**Métodos** Cinquenta e uma mulheres grávidas consecutivas com uma única gravidez entre a 24ª e a 36ª semanas com diagnóstico de ameaça de trabalho de parto prematuro foram incluídas no presente estudo de corte prospectivo.

**Resultados** Como resultado da análise de regressão logística multivariada para prever parto prematuro dentro de 24 horas, 48 horas, 7 dias, 14 dias, ≤ 35 semanas gestacionais e ≤ 37 semanas gestacionais após a admissão, área sob a curva (AUC) (95% de confiança os valores de intervalo [CI[) foram 0,95 (0,89–1,00), 0,93 (0,86–0,99), 0,91 (0,83–0,98), 0,92 (0,85–0,99), 0,82 (0,69–0,96) e 0,89 (0,80–0,98), respectivamente. No presente estudo, os níveis de IMA e biglican foram maiores e os níveis de decorin menores em mulheres admitidas no hospital com ameaça de trabalho de parto prematuro e que tiveram parto prematuro em 48 horas em comparação com aquelas que deram à luz após 48 horas.

**Conclusão** Em gestantes admitidas no hospital com ameaça de trabalho de parto prematuro, a predição de parto prematuro do modelo combinado criado pela adição de IMA, decorin e biglican, além da medição do TVS CL, foi maior do que a medição do TVS CL isoladamente.

**Registro do ensaio clínico** O presente ensaio foi registrado em ClinicalTrials.gov, número NCT04451928.

## Introduction

Births occurring after the 20 ^th^ week of pregnancy and before the 37 ^th^ week are called preterm delivery. It has been reported by the World Health Organization (WHO) that 9.6% of all births are preterm deliveries. 1 Preterm labor is one of the most important causes of infant mortality and morbidity. Risk factors for preterm delivery include systemic and genital tract infections, periodontal disease, reduced cervical length, previous cervical surgeries, congenital abnormalities of the uterus, smoking and substance abuse, nutritional deficiency, black race, low socioeconomic level, low educational level, genetic predisposition to preterm delivery, having a premature birth, and multiple pregnancies. 2 Unfortunately, half of preterm deliveries occur in pregnant women without any risk factors. Numerous studies have been conducted in the literature to predict preterm birth in women in threatened preterm labor. However, there is no single or combined screening method for high-sensitivity preterm birth to clearly identify women at risk of preterm birth. 3 4 5 6 7 8 9 10 Current markers give low predictions of which pregnancies will have preterm delivery. 11 12 The unclear pathogenesis contributes to the unpredictability. 13 The most cited mechanisms include premature activation of the maternal or fetal hypothalamic-pituitary-adrenal axis, inflammation and infection, decidual hemorrhage, and pathological uterine distension. Forty to 45% of the PTB cases are idiopathic (spontaneous). Previous preterm birth, maternal nutritional status, presence of infection or inflammation, and various demographic factors such as age and race are important risk factors for spontaneous PTB. Infection and/or inflammation are thought to play a role in ∼ 30% of spontaneous PTB cases. 14 Despite an unproven link between vaginal microbiology and PTB, an abundant body of literature exists on the subject. Bacterial vaginosis, increased colonization of F. nucleatum, Mycoplasma hominis, Bacteriodes urealytocius and the loss of Lactobacillus species are some of the proposed mechanisms between the change in vaginal microbiome and PTB. 15 16 In a recent study, it was shown that BV-associated bacterium 1(BVAB1), *Prevotella* cluster 2, *S. amnii* and TM7-H1, and other taxa may have roles in the etiology of PTB. 17

Cervical length measurement by transvaginal sonography (TVS CL) is one of the most common tests to predict preterm delivery. Knowledge of cervical length in women with threatened preterm labor may improve outcome but data are limited. 18

Albumin is abundant in human plasma and acts as a buffer for toxic molecules. The N-terminus of albumin binds nucleic acids, lipids, other proteins, and metals. In ischemia, the structure of albumin changes. When ischemia develops, free oxygen radicals emerge in the environment and damage the N terminus of albumin. It becomes difficult for albumin affected by ischemia to bind divalent metals in the N-terminus, 19 and this new molecule whose structure has changed is called ischemia-modified albumin (IMA). 20

Ischemia modified albumin is used in cardiac ischemic diseases to determine the early stages of ischemia in which necrosis has not yet occurred. It has been claimed that it increases in the early stages in response to ischemia and will prevent the progression of myocardial damage. It has been shown that IMA levels are higher in pregnant women compared with nonpregnant women. 21 Also, IMA increases in cases where placental perfusion is impaired during pregnancy and oxidative stress and inflammation increase. 22 In cases of increased oxidative stress where this balance cannot be achieved, it may cause pathologies such as pre-eclampsia, intrauterine growth restriction (IUGR), preterm labor, and spontaneous abortion. 23 Ischemia modified albumin increases in pregnancies complicated by early pregnancy loss, 22 recurrent pregnancy loss, 24 hyperemesis gravidarum, 25 gestational diabetes, 26 27 pre-eclampsia, 28 small for gestational age (SGA) fetuses ^2^ 9 and IUGR. 30 However, there is no study investigating the significance of IMA in preterm labor. It is proposed that the oxidative stress and inflammation are related to the pathogenesis of preterm birth in various studies. 31 Increase of IMA in preterm birth seems to be related to the increase of oxidative stress and inflammation in preterm birth rather than having a role in the pathogenesis of preterm birth.

Biglycan and decorin are proteoglycans found in the intermediate and reticular layers of human fetal membranes. 32 These proteoglycans form the extracellular matrix. The extracellular matrix increases the tensile strength of connective tissue. 33 34 It stabilizes the architecture of tissues by binding to decorin collagen fibres. 33 34 35 36 Biglycan destabilizes the decorin-collagen relationship. 34 35 During the 3 ^rd^ trimester of pregnancy and active labor, the ratio of biglycan to decorin increases in fetal membranes. This increased rate is thought to contribute to the mechanical weakening of the membranes. 37 Premature rupture of fetal membranes (PPROM) was observed in the 2 ^nd^ trimester of pregnancy in asymptomatic pregnant women with increased serum biglycan levels in the following weeks of pregnancy. Also, it was found that while biglycan was high in these pregnant women, serum decorin levels decreased. 38 In mouse studies, in models with genetic mutations and lack of informative or decorin, when biglycan decreases, decorin was found to be higher. 39 It is thought that these two molecules are compensating for each other. However, this coordination could not be demonstrated in human fetal membranes. 24 The relationship between increased decorin levels and decorin to biglycan ratio and the increase of these in maternal serum has not been explained in the literature. Uzun Cilingir et al. found increased maternal serum and placental tissue levels of pre-eclamptic women in their study, which included women in the 3 ^rd^ trimester. Although a correlation analysis has not been performed for the placental and maternal serum decorin levels in this study, it is shown that both increase concurrently. 40 However, this relationship has not been performed yet for biglycan and decorin to biglycan ratio in the literature. Probably, increased decorin levels in membranes, hence the increase in decorin to bigylcan ratio in membranes, are possibly reflected and to amniotic fluid and passage to the maternal serum during preterm birth.

In the present study, the levels of IMA, biglycan, and decorin in the serum of pregnant women hospitalized for threatened preterm labor (preterm delivery within 24 hours, 48 hours, 7 days, 14 days, ≤ 35 gestational weeks, and ≤ 37 gestational weeks after admission) were examined. Serum levels were compared between women having preterm and term delivery.

## Methods

The present prospective cohort study was conducted between December 2019 and December 2020 at the Evliya Çelebi Training and Research Hospital of the Kütahya Health Sciences University. Ethics committee approval was obtained prior to the study (2019-01/1). Informed consent was obtained from every patient included in the study. The present trial was registered at ClinicalTrials.gov, number NCT04451928

Fifty-one consecutive pregnant women aged between 18 and 42 years old who were hospitalized with a diagnosis of threatened preterm labor and who had a singleton pregnancy between the 24 ^th^ and 36 ^th^ 6 weeks were included in the present study. Women were also enrolled only if they had intact amniotic membrane, uterine contraction ≥ 3 times in 30 minutes, cervical dilatation < 3 cm, and cervical effacement < 80%. 9 Women were excluded if they had multiple pregnancies, PPROM, abnormal placentation (such as placenta previa), uterine anomaly, maternal heart disease, inflammatory or infectious disease, pre-eclampsia, fetal growth restriction, congenital fetal anomaly, polyhydramnios, acute chorioamnionitis, and medically-induced preterm delivery.

Patients admitted to the hospital due to threatened preterm labor primarily received bed rest and hydration. When cervical changes persisted or contractions continued after 2 hours after intravenous hydration, tocolytic treatment was started. Calcium channel blockers were used as a tocolytic drug when needed. Maternal corticosteroid (12 mg intramuscular betamethasone within 24 hours) was given when needed to accelerate fetal lung development. Forty-eight hours after the steroid administration, tocolysis was stopped. Demographic data of the patients were recorded. Patients were followed until delivery. The gestational week was determined according to the last menstrual date and confirmed by early ultrasonographic measurements. The gestational week at birth and the time between admission to the hospital and birth were recorded. Delivery time was divided into groups as preterm delivery within 24 hours, 48 hours, 7 days, 14 days, ≤ 35 gestational weeks, and ≤ 37 gestational weeks after admission. 41 42 43 44 45 Mode of delivery, birth weight and APGAR score were recorded.

Spontaneous preterm labor (sPTL) leading to PTB is a heterogeneous condition, with a multifactorial etiology. Various different mechanisms with different pathways, including increased contractility, membrane rupture, and cervical changes leads to preterm birth. 46 Due to its multifactorial nature, it has not been possible to predict sPTL and PTB with a single marker. So, combinations of various markers were evaluated in similar prediction studies. That is why we also tried to use a combination of several different markers each concerning different etiopathogenetic pathways. Our proposed model and the serum markers used in our study are not in daily clinical use in predicting threatened preterm delivery. Additionally, we do not claim the clinical use of our results in until future stronger studies support our results.

Clinically available predictive methods for women with symptoms of preterm labor are sonographic transvaginal cervical length (CL) measurement and bedside biomarker tests in cervical/vaginal fluid, such as fetal fibronectin (fFN), phosphorylated insulin-like growth factor binding protein-1 (phIGFBP-1 or Actim Partus), or placental alpha microglobulin-1 (PAMG-1 or Partosure). 47 However, the utility of these tests has not been validated in either large or randomized clinical trials.

Similar to our study, studies in the literature using various combinations of serum or vaginal biomarkers with CL measurement for prediction of preterm delivery in threatened preterm labor also performed serum biomarker measurements as early as possible at the time of diagnosis of threatened preterm birth. 48

Cervical length measurement by transvaginal sonography during evaluation for preterm labor symptoms was measured with a 4 to 10 MHz transvaginal probe (Toshiba Medical Systems Corporation, Japan) with an empty bladder. Research personnel performing transvaginal CL measurement were trained, and all images were reviewed for adequacy and accuracy using the protocol described by Iams et al. at the time of image ascertainment. 49 The shortest CL measurement was used for each patient.

Blood withdrawal for serum biomarkers in our study was performed as soon as the threatened labor diagnosis was confirmed when uterine contractions with cervical changes persisted after 2 hours of bed rest and hydration.

Venous blood samples were taken from the antecubital vein of the patients. Blood samples were transferred to non-heparinized tubes. The tubes were centrifuged at 1,500 xg for 10 minutes. Serum samples obtained afterwards were stored in a freezer at - 80°C until analysis.

Levels of serum IMA were assayed with an ELISA kit (Human [IMA] ischemia modified albumin, Cat. No: E-EL-H5422, Elabscience, Texas, USA). Results were expressed as ng per mL of serum (ng/mL). The sensitivity of this kit was 1.88 ng/mL. Intra- and inter-CV were 5.2 and 6.4%, respectively.

Levels of serum DCN were assayed with an ELISA kit (Human [DCN] decorin, Cat. No: E-EL-H2248, Elabscience, Texas, USA). Results were expressed as ng per mL of serum (ng/mL). The sensitivity of this kit was 0.75 ng/mL. Intra- and inter-CV were 5.4 and 6.7%, respectively.

Levels of serum BGN were assayed with an ELISA kit (Human [BGN] biglycan, Cat. No: E-EL-H6091, Elabscience, Texas, USA). Results were expressed as pg per mL of serum (pg/mL). The sensitivity of this kit was 18.75 pg/mL. Intra- and inter-CV were 5.3 and 6.2%, respectively.

For data analysis, the IBM SPSS Statistics for Windows version 21.0 (IBM Corp., Armonk, NY, USA) and R statistical computing software (version 3.6.1, https://www.r-project.org/ ) were used. Data are presented as mean ± standard deviation (SD) and median (25 ^th^ percentile; 75 ^th^ percentile). Conformity to normal distribution was evaluated with the Shapiro-Wilk or the Kolmogorov-Smirnov test. Quantitative data of the groups were compared with the Student t-test or the Mann-Whitney U test. Univariate logistic regression analyses were performed to determine associations between each individual marker and preterm delivery. Multivariate logistic regression analysis of candidate serum biomarkers along with CL was performed to determine a combined model for predicting preterm delivery within 24 hours, 48 hours, 7 days, 14 days, ≤ 35 gestational weeks, and ≤ 37 gestational weeks after admission. Receiver operating characteristic (ROC) curve analysis was performed to calculate the area under the curve (AUC) values for the different markers and the combined model. A value of *p* < 0.05 was considered statistically significant.

## Results

Forty-nine percent (26/51) of the threatened preterm labor cohort group resulted in preterm delivery (< 37 weeks). Characteristics of the study population of threatened preterm labor are shown in Table 1 . A total of 29.4% (15/51) of the newborns needed neonatal intensive care. A total of 39.2% (20/51) of the newborns were female. A total of 56.9% (29/51) of the deliveries were performed vaginally. There was a history of preterm delivery in 29.4% (15/51) of the cases.

**Table 1:** Characteristics of the study population of threatened preterm labor ( *n* = 51)

Age (years old)	29.13 ± 6.65
	29.00 [23.0–34.0]
BMI (kg/m2)	22.72 ± 3.66
	22.6 [20.3–25.0]
Gravidity	2.27 ± 1,11
	2 [1–3]
Parity	1.01 ± 0.88
	1 [0–2]
Gestational age (weeks)	34.23 ± 2.71
	34.86 [33.43–36.29]
IMA (ng/mL)	43.17 ± 14.08
	50.53 [34.34–49.07]
Decorin (ng/mL)	14.87 ± 6.27
	12.65 [9.53–18.73]
Biglycan (pg/mL)	465.07 ± 175.68
	435.0 [382.0–477.0]
Cervical length (mm)	30.81 ± 6.84
	32.0 [28.0–35.2]
Gestational age at delivery (weeks)	36.94 ± 2.70
	37.14 [36.29–39.0]
Newborn weight (g)	2951.09 ± 552.30
	3130.0 [2710.0–3280.0]
Steroid/tocolytic administration	50.98% (26/51)
Neonatal intensive care need	39.2% (20/51)

Abbreviations: BMI, body mass index; IMA, ischemia modified albumin.

Data are presented as mean ± standard deviation, median [interquartile range] or number (percentage).

In the present study, IMA and biglycan levels were found to be higher and decorin levels were lower in women admitted to the hospital with threatened preterm labor and who had a preterm delivery within 48 hours compared to preterm delivery after 48 hours (respectively, *p* = 0.043, *p* = 0.029, and *p* = 0.014). Diagnostic indices of three candidate protein biomarkers, CL, and the final combined model for predicting preterm delivery within 24 hours, 48 hours, 7 days, 14 days, ≤ 35 weeks of gestation, and ≤ 37 weeks of gestation women with threatened preterm labor in the total cohort are shown in Table 2 and Table 3 .

**Table 2:** Diagnostic indices of three candidate protein biomarkers, cervical length and the final combined model for predicting spontaneous preterm birth within 24 hours, 48 hours, and 7 days with preterm labor in the total cohort

	24 hours			48 hours			7 days		
Preterm birth ratio	25.5% (13/51)			29.4% (15/51)			37.3% (19/51)		
	OR (95%CI)	*p-value*	AUC (95%CI)	OR (%95CI)	*p-value*	AUC (95%CI)	OR (%95CI)	*p-value*	AUC (95%CI)
IMA	1.07 (1.02–1.15)	0.014*	0.69 (0.51–0.872)	1.07 (1.02–1.14)	0.014*	0.68 (0.50–0.86)	1.07 (1.02–1.15)	0.013*	0.68 (0.52–0.84)
Decorin	0.88 (0.75–1.00)	0.080	0.67 (0.51–0.83)	0.85 (0.72–0.97)	0.032*	0.72 (0.57–0.8679)	0.85 (0.73–0.95)	0.014*	0.73 (0.59–0.87)
Biglycan	1.01 (1.00–1.02)	0.019*	0.74 (0.56–0.91)	1.01 (1.00–1.02)	0.024*	0.70 (0.52–0.87)	1.01 (1.00–1.02)	0.019*	0.71 (0.56–0.87)
Cervical Length	0.80 (0.67–0.90)	0.002*	0.89 (0.80–0.98)	0.82 (0.70–0.92)	0.003*	0.85 (0.75–0.96)	0.85 (0.74–0.94)	0.007*	0.80 (0.67–0.93)
*Combined model 1*									
IMA	1.11 (1.01–1.25)	0.048*	0.88 (0.78–0.98)	1.09 (1.01–1.21)	0.059	0.86 (0.76–0.96)	1.10 (1.01–1.22)	0.043*	0.88 (0.78–0.97)
Decorin	0.84 (0.63–1.03)	0.157		0.81 (0.61–0.98)	0.069		0.82 (0.65–0.97)	0.043*	
Biglycan	1.02 (1.01–1.04)	0.028*		1.01 (1.00–1.03)	0.033*		1.02 (1.01–1.03)	0.015*	
*Combined model 2*									
IMA	1.17 (1.03–1.42)	0.039*	0.95 (0.89– 1)	1.13 (1.02–1.29)	0.040*	0.93 (0.86–0.99)	1.12 (1.02–1.25)	0.031*	0.91 (0.83–0.98)
Decorin	0.71 (0.47–0.93)	0.042*		0.74 (0.55–0.93)	0.022*		0.79 (0.61–0.95)	0.025*	
Biglycan	1.02 (1.00–1.04)	0.100		1.01 (1.00–1.03)	0.161		1.01 (1.00–1.03)	0.061	
Cervical Length	0.65 (0.41–0.86)	0.018*		0.74 (0.55–0.91)	0.016*		0.83 (0.67–0.98)	0.044*	

Abbreviations: AUC, area under the curve; CI, confidence interval; IMA, ischemia modified albumin; OR, odds ratio.

Statistically significant comparisons were marked with *.

**Table 3:** Diagnostic indices of three candidate protein biomarkers, cervical length, and the final combined model for predicting spontaneous preterm birth within 14 days of sampling of and before 35 weeks of gestation, before 37 weeks of gestation women with preterm labor in the total cohort

	14 days			< 35 weeks			< 37 weeks		
Preterm birth ratio	45.1% (23/51)			23.5% (12/51)			51.0% (26/51)		
	OR (95%CI)	*p-value*	AUC (95%CI)	OR (95%CI)	*p-value*	AUC (95%CI)	OR (95%CI)	*p-value*	AUC (95%CI)
IMA	1.07 (1.02–1.15)	0.013*	0.67 (0.52–0.82)	1.01 (0.96–1.05)	0.827	0.51 (0.31–0.71)	1.04 (1.00–1.10)	0.085	0.60 (0.44–0.76)
Decorin	0.85 (0.73–0.95)	0.014*	0.72 (0.57– 0.86)	0.93 (0.81–1.04)	0.245	0.61 (0.43–0.79)	0.87 (0.77–0.96)	0.012*	0.71 (0.56–0.86)
Biglycan	1.01 (1.00–1.02)	0.019*	0.73 (0.59–0.88)	1.00 (1.00–1.01)	0.271	0.70 (0.54–0.86)	1.01 (1.00–1.02)	0.017*	0.71 (0.57–0.85)
Cervical Length	0.85 (0.74–0.94)	**0.007**	0.83 (0.72–0.95)	0.88 (0.78–0.97)	0.014*	0.82 (0.70–0.93)	0.77 (0.63–0.89)	0.002*	0.81 (0.68–0.93)
*Combined model 1*									
IMA	1.08 (1.01–1.18)	0.060	0.86 (0.76–0.96)	0.98 (0.92–1.04)	0.503	0.69 (0.50–0.88)	1.03 (0.97–1.10)	0.386	0.82 (0.70–0.94)
Decorin	0.85 (0.71–0.99)	0.051		0.92 (0.80–1.04)	0.215		0.88 (0.76–0.99)	0.046*	
Biglycan	1.02 (1.01–1.03)	0.008*		1.00 (1.00–1.01)	0.213		1.01 (1.00–1.02)	0.020*	
*Combined model 2*									
IMA	1.12 (1.02–1.25)	0.031*	0.92 (0.85–0.99)	0.99 (0.93–1.05)	0.719	0.82 (0.69–0.96)	1.04 (0.96–1.14)	0.319	0.89 (0.80–0.98)
Decorin	0.79 (0.61–0.95)	0.025*		0.91 (0.78–1.04)	0.193		0.85 (0.71–0.99)	0.047*	
Biglycan	1.01 (1.00–1.03)	0.061		1.00 (0.99–1.00)	0.966		1.01 (1.00–1.02)	0.192	
Cervical Length	0.83 (0.67–0.98)	0.044*		0.87 (0.76–0.97)	0.020*		0.81 (0.67–0.94)	0.012*	

Abbreviations: AUC, area under the curve; CI, confidence interval; IMA, ischemia modified albumin; OR, odds ratio.

Statistically significant comparisons were marked with *.

Serum IMA level was found to be significant in predicting preterm delivery within 24 hours, 48 hours, 7 days, and 14 days after admission as a result of multivariate logistic regression analysis (respectively, *p* = 0.039, *p* = 0.040, *p* = 0.031, and *p* = 0.031). Decorin level was significant in predicting preterm delivery within 24 hours, 48 hours, 7 days, 14 days, and ≤ 37 gestational weeks after admission (respectively, *p* = 0.042, *p* = 0.022, *p* = 0.025, *p* = 0.025, and *p* = 0.047). Biglycan level was insignificant in predicting preterm delivery within 24 hours, 48 hours, 7 days, 14 days, ≤ 35 gestational weeks, and ≤ 37 gestational weeks after admission ( *p* > 0.05). Cervical length was significant in predicting preterm delivery within 24 hours, 48 hours, 7 days, 14 days, ≤ 35 gestational weeks, and ≤ 37 gestational weeks after admission ( *p* = 0.018, *p* = 0.016, *p* = 0.044, *p* = 0.044, *p* = 0.020, and *p* = 0.012, respectively). Area under the curve values of the final combined model 1 (3 biochemical markers) for predicting preterm delivery within 24 hours, 48 hours, 7 days, 14 days, ≤ 35 gestational weeks, and ≤ 37 gestational weeks after admission were 0.88 (0.78–0.98), 0.86 (0.76–0.96), 0.88 (0.78–0.97), 0.86 (0.76–0.96), 0.69 (0.50–0.88), and 0.89 0.81 (0.68–0.93), respectively. Area under the curve values of the final combined model 2 (CL plus other 3 biochemical markers) for predicting preterm delivery within 24 hours, 48 hours, 7 days, 14 days, ≤ 35 gestational weeks, and ≤ 37 gestational weeks after admission were 0.95 (0.89–1.00), 0.93 (0.86–0.99), 0.91 (0.83–0.98), 0.92 (0.85–0.99), 0.82 (0.69–0.96), and 0.89 (0.80–0.98), respectively.

## Discussion

In the present study, IMA and biglycan levels were higher and decorin levels were lower in women admitted to the hospital with threatened preterm labor and who had a preterm delivery within 48 hours compared with those who gave birth after 48 hours (respectively, *p* = 0.043, *p* = 0.029, and *p* = 0.014). In predicting the diagnostic indices of the final combined model (3 candidate protein biomarkers plus CL) for predicting preterm delivery within 24 hours, 48 hours, 7 days, 14 days, ≤ 35 gestational weeks, and ≤ 37 gestational weeks after admission, prediction AUC (95% confidence interval [CI]) values were 0.95 (0.89–1.00), 0.93 (0.86–0.99), 0.91 (0.83–0.98), 0.92 (0.85–0.99), 0.82 (0.69–0.96), and 0.89 (0.80–0.98), respectively.

Numerous studies have been conducted in the literature to predict preterm birth in women in threatened preterm labor. However, there is no single or combined screening method for high-sensitivity preterm birth to clearly identify women at risk of preterm birth. 3 4 5 6 7 8 9 10 In the previously conducted studies, it was shown that while the levels of biglycan increase in fetal membranes after labor, decorin levels decrease. 37 Atalay et al. found that serum decorin has a limited effect in the prediction of preterm delivery within 1 week or before 34 weeks. But a combination of serum decorin with CL measurements predicted preterm delivery before 37 weeks. 43 In the study by Underhill and et al., patients with PPROM had high serum biglycan levels and low decorin levels. 38 In the present study, in the univariate analysis, similar to Underhill et al., decorin and biglycan were found to be significant in predicting preterm delivery. However, in the multivariate analysis, biglycan was not significant in predicting preterm delivery. In the study by Atalay et al., pregnant women with 24 to 32 weeks of gestation are included, similarly to our study. 43 However, their study was a case control study, unlike ours, which we designed as a cohort study which makes it not right to make comparison between studies.

In the study by Underhill et al. (which is a retrospective case control study), PPROM risk was tried to be predicted by the serum biglycan and decorin levels in 15 to 20 weeks of pregnancy. 38 They found an AUC value of 0.659 for biglycan and 0.563 for decorin. However, in our study, AUC values range between 0.69 and 0.73 for biglycan and between 0.61 and 0.87 for decorin for 5 different primary outcomes, as can be seen in Table 2 . Thus, the study design of Underhill et al. and ours differ considerably, which makes it not right to make comparison between studies. The reason that our AUC values are higher than the values in the study of Underhill et al. is that our cohort consists of women with threatened preterm labor.

Cervical length measurement by transvaginal sonography is one of the most common tests to predict preterm delivery. Knowledge of cervical length in women with threatened preterm labor may improve outcome but data are limited. 18 Ness et al. stated in their study that > 50% of pregnant women who were admitted to the hospital with threatened preterm labor and who had TVS CL ≥ 30 mm were discharged and the probability of delivery within 7 days after admission was < 2%. 50 In the literature, it was aimed to increase the prediction rates of preterm delivery by adding markers to the TVS CL measurement to determine the risk of preterm delivery in pregnant women presenting with threatened preterm labor. However, in routine clinical practice, there is so far no solid marker in addition to TVS CL measurement to determine preterm delivery risk in symptomatic women with threatened labour. 43 In the present study, the AUC values of the TVS CL measurement in preterm delivery prediction were > 0.8. In addition to the TVS CL measurement, the preterm delivery prediction of the combined model, which was created by adding IMA, decorin, and biglycan, was higher than the TVS CL measurement alone.

In addition to TVS CL measurement, the most investigated measurement in the literature is fetal fibronectin. Although many studies implicated the role of fetal fibronectin in vaginal secretions in prediction of preterm delivery in symptomatic women, routine clinical use has not gained widespread use. Fetal fibronectin testing in singleton gestations with threatened preterm labor is not associated with the prevention of preterm birth or improvement in perinatal outcome but is associated with higher costs. 51 In addition to the cost and questionable effectiveness, fetal fibronectin results may be affected by coitus within 48 hours preceding testing. 52

An AUC value of 0.78 was determined in predicting preterm delivery before 34 weeks using quantitative fetal fibronectin for symptomatic high-risk women in a large prospective study. 52 In another prospective study, the AUC was 0.95 using a model combining TVS CL measurement with fetal fibronectin in symptomatic cases. 51 Our combined model using three serum biochemical markers in addition to TVS CL had nearly the same AUC value.

In a recent meta-analysis, the AUC for predicting preterm delivery at ≤ 7 days for placental alpha microglobulin-1 (PAMG-1), fetal fibronectin (fFN) and insulin-like growth factor-binding protein-1 (phIGFBP-1) were 0.961, 0.874, and 0.801, respectively, in symptomatic women. 53 In a recent study, using an application (QUiPP App prototype) that uses fetal fibronectin and TVS CL measurement for the prediction of preterm delivery, AUC values were 0.96, 0.85, 0.77, 0.91, and 0.92 for preterm delivery < 30 weeks, < 34 weeks, < 37 weeks, < 1 week, and < 2 weeks, respectively. 54 Although we obtained lower AUC values for each single marker, our combined model reached an AUC of 0.95, which is compatible with the highest values in the relevant literature. However, we are aware that our findings need to be substantiated given the small number of subjects.

As a result, IMA and biglycan levels were found to be higher and decorin levels lower in women admitted to the hospital with threatened preterm labor and who had preterm birth within 48 hours compared with those who gave birth after 48 hours. Preterm delivery prediction of the combined model created by adding IMA, decorin, and biglycan in addition to the TVS CL measurement in pregnant women presenting with threatened preterm labor was higher than the TVS CL measurement alone for all women in the present study. The results show that serum IMA, decorin, and biglycan concentrations and the TVS CL measurement may be a useful marker for monitoring preterm delivery in symptomatic women.

The smaller case number is the major limitation of our study. Additionally, it needs to be noted that the predictive performance and utility of the test would be different if the concept of our study was to predict preterm birth by measuring these serum biochemical markers and CL in the 2 ^nd^ trimester before the threatened preterm labor has taken place. We believe that an important contribution to the literature for predicting preterm labor can be made if our parameters could be studied in a low-risk population during the 2 ^nd^ trimester.
